# 25-Hydroxyvitamin D3-Deficiency Enhances Oxidative Stress and Corticosteroid Resistance in Severe Asthma Exacerbation

**DOI:** 10.1371/journal.pone.0111599

**Published:** 2014-11-07

**Authors:** Nan lan, Guangyan Luo, Xiaoqiong Yang, Yuanyuan Cheng, Yun zhang, Xiaoyun Wang, Xing Wang, Tao Xie, Guoping Li, Zhigang Liu, Nanshan Zhong

**Affiliations:** 1 Inflammations & Allergic Diseases Research Unit, Affiliated Hospital of Luzhou Medical College, Luzhou, 646000, Sichuan, China; 2 Hygiene Section, Luzhou Medical College, Luzhou, 646000, Sichuan, China; 3 State Key Laboratory of Respiratory Disease for Allergy at Shengzhen University, School of Medicine, Shenzhen University, Nanhai Ave 3688, Shenzhen, Guangdong, 518060, PR China; 4 State Key Laboratory of Respiratory Disease, Guangzhou Medical University, Guangdong, 510120, PR China; University of Missouri-Kansas City, United States of America

## Abstract

Oxidative stress plays a significant role in exacerbation of asthma. The role of vitamin D in oxidative stress and asthma exacerbation remains unclear. We aimed to determine the relationship between vitamin D status and oxidative stress in asthma exacerbation. Severe asthma exacerbation patients with 25-hydroxyvitamin D3-deficiency (V-D deficiency) or 25-hydroxyvitamin D-sufficiency (V-D sufficiency) were enrolled. Severe asthma exacerbation with V-D-deficiency showed lower forced expiratory volume in one second (FEV1) compared to that with V-D-sufficiency. V-D-deficiency intensified ROS release and DNA damage and increased TNF-α, OGG1 and NFκB expression and NFκB phosphorylation in severe asthma exacerbation. Supplemental vitamin D3 significantly increased the rates of FEV1 change and decreased ROS and DNA damage in V-D-deficiency. Vitamin D3 inhibited LPS-induced ROS and DNA damage and were associated with a decline in TNF-α and NFκB in epithelial cells. H_2_O_2_ reduces nuclear translocation of glucocorticoid receptors in airway epithelial cell lines. V-D pretreatment enhanced the dexamethasone-induced nuclear translocation of glucocorticoid receptors in airway epithelial cell lines and monocytes from 25-hydroxyvitamin D3-deficiency asthma patients. These findings indicate that V-D deficiency aggravates oxidative stress and DNA damage, suggesting a possible mechanism for corticosteroid resistance in severe asthma exacerbation.

## Background

Severe asthma is unresponsive to treatment, including systemically administered corticosteroids [1]. Asthma exacerbation results in oral steroid use, an emergency room visit, or hospitalization [2]. Severe asthma is defined as asthma that requires treatment with high dose inhaled corticosteroids plus a second controller and/or systemic corticosteroids to prevent it from becoming "uncontrolled" or that remains "uncontrolled" despite this therapy [3]. The multiple risk factors for asthma exacerbation include a complex mix of environmental, immunological and host genetic factors. Epidemiological studies have shown that low serum 25-hydroxyvitamin D3 levels are associated with a higher risk of upper and lower respiratory infections [4]. Vitamin D status has a linear relationship with respiratory infections and lung function [5], and V-D deficiency (a serum 25 (OH) D3 <30 ng/ml) has been associated with severe asthma exacerbation [6].

An endotoxin is a common environmental contaminant that causes asthma exacerbation [7]. Environmental endotoxins modulate the exacerbation of asthma. High levels of endotoxins are associated with asthma symptoms and current use of asthma medication. Endotoxins augment atopic inflammation and induce cellular steroid resistance [8], [9]. Corticosteroid-resistant asthma demonstrates the airway expansion of specific gram-negative bacteria [10]. The underlying mechanisms by which endotoxins modulate asthma are not completely understood. Oxidative stress is an important aspect of the host innate immune response to foreign pathogens such as bacterial lipopolysaccharides (LPS). Excessive activation of redox signaling might lead to pathologic endothelial cell (EC) activation and barrier dysfunction [11]. Invading pathogens might result in overwhelming lung production of reactive oxygen species (ROS). Oxidants initiate inflammation of the airways, which might contribute to the pathogenesis and/or exacerbation of airway diseases [12]. Oxidative stress is associated with DNA damage, which is frequently induced by ROS released from neutrophils or respiratory tract epithelial cells [13].

It has recently been shown that an imbalance between oxidants and antioxidants is associated with oxidative stress, which plays a key role in the severity of asthma [14]. One study shows that Vitamin D supplementation could replenish blood levels and lower oxidative stress and cardiovascular disease [15]. Vitamin D exhibited therapeutic and preventive effects against oxidative stress, hepatic, pancreatic and renal injury in alloxan-induced diabetes in rats [16]. Vitamin D played a role against cellular stress in breast epithelial cells [17]. However, whether oxidative stress and DNA damage are associated with asthma exacerbation and the vitamin D status is unknown. To address this question, we studied the effect of the vitamin D status on oxidative stress and DNA damage in patients with severe asthma exacerbation and in LPS-stimulated cells.

## Methods

### Subjects

Asthma patients with acute exacerbation were admitted to the respiratory department of the affiliated hospital of Luzhou medical college (a 3000-bed hospital in Luzhou City, Sichuan, China), Luzhou, China, between September 2011 and December 2012. Acute exacerbation of asthma was defined as a worsening of asthma symptoms including shortness of breath, cough, wheezing, chest tightness, or a combination of these symptoms. Severe asthma exacerbations were defined as those that required treatment with systemic corticosteroids [18]. Patients with a radiological diagnosis of pneumonia, impaired consciousness on admission or smoking history >10 packs/year were excluded from the study. The serum levels of 25-hydroxyvitamin D3 (vitamin D3) were measured at the beginning of the study. We categorized this measurement into deficiency (≤30 ng/mL) and sufficiency (>30 ng/mL) categories based on a previous report [19]. The forced expiratory volume in one second (FEV1) was measured with a Jaeger Lung Function Analyzer (Jaeger Co., H chberg, Germany).

### Ethics statement

The study was approved by the Ethics Committee of the Affiliated Hospital of Luzhou Medical College (KY2013014). The participants provided their written informed consent to participate in this study. The Ethics Committee of the Affiliated Hospital of Luzhou Medical College approved the use of written consent.

### Therapy of asthma exacerbations

Asthmatic patients with severe asthma exacerbation were treated with 80 mg/day of methylprednisolone for 7 days [18]. The asthma patients with V-D-deficiency were randomly divided into V-D-supplement group and no V-D-supplement group. The asthma patients with V-D-deficiency in V-D-supplement group were intramuscularly injected with 7.5 mg vitamin of D3 at day 1 and 4.

### Measurement of vitamin D3, TNF-α and SOD in serum

The serum vitamin D3 and tumor necrosis factor-α (TNF-αwere determined in triplicate samples from each patient by enzyme-linked immunosorbent assay (ELISA). The ELISA kits for TNF-α were purchased from R&D Systems (Minneapolis, MN). The ELISA kits for vitamin D3 were purchased from IBL (Germany). The superoxide dismutases (SOD) activity in serum was measured using the SOD assay kit WST (Nanjing Jiancheng Bioengineering Institute, China).

### Mononuclear cell separation

The peripheral blood obtained from the asthma patients and normal donors was processed for separation of mononuclear cells. Peripheral blood was diluted 1∶1 with sterile phosphate-buffered saline (PBS), layered over Ficoll-Hypaque (GE Healthcare Bio-Sciences AB, Stockholm, Sweden) and centrifuged at 1500 rpm for 15 min at room temperature. Peripheral blood mononuclear cells (PBMC) were collected from the interphase layer and washed with PBS. PBMC were resuspended with RPMI-1640 medium.

### Airway epithelial cells culture

After the human airway epithelial cells (16HBE) had grown to 85% confluence in 6-well plastic plates containing DMEM/F12 culture medium with 10% of foetal calf serum (FCS), the medium was replaced with a serum-free DMEM/F12 culture medium. The cells were then treated with LPS and/or 100 nM 1, 25(OH) 2 D3 (CAT. # 083M4033V, Sigma) in a serum-free culture medium.

### Measurement of ROS in PBMC and cultured epithelial cells

The cells were loaded with 10 µM H2DCF-DA (Invitrogen, Molecular Probes, USA) or 10 µM dihydroethidium (DHE) at 37°C for 30 minutes according to the manufacturer's instructions. After removing the excess probes, the cells were kept at 37°C with 5% CO_2_. The fluorescence intensity was detected by a flow cytometer (Beckman Coulter, US) and a Leica TCS SP5 confocal microscope (Leica, Germany). For each sample, 10,000 events were collected.

### Measurement of DNA damage in PBMC and cultured epithelial cells

A comet assay was used to detect the DNA damage and was performed as previously described [20]. A total of 10 µl of cell suspension containing 20,000 cells was mixed with 90 µl of low-melting-point agarose (LMA) (Sigma) in PBS at 37°C and layered onto slides, which had been coated with normal melting point agarose. The slides were submersed in a freshly prepared cold (4°C) lysis solution (2.5 M NaCl, 100 mM EDTA-2Na, 10 mM Tris–HCl at pH 10-10.5, 1% Triton X-100 and 10% DMSO) for 2 hours. The slides were immersed in fresh electrophoresis buffer at 4°C for 30 min and then electrophoresed (25 V/300 Ma) for 25 min. After electrophoresis, the slides were stained with ethidium bromide, covered with a coverslip and analyzed using a Leica TCS SP5 confocal microscope (Leica, Germany). Comet Assay IV software was used to assess the DNA damage score.

### Immunocytochemistry

PBMCs were incubated with dexamethasone (1 µM) or 1, 25(OH) 2 D3 (100 nM) for 30 minutes. PBMCs were immunostained for glucocorticoid receptor antibodies (1:500 dilution) (sc-1004, Santa Cruz Biotechnology). Tetramethylrhodamine isothiocyanate (TRITC)–conjugated anti-rabbit secondary Abs or fluorescein isothiocyanate (FITC)-were used to probe the primary Abs. The nucleus was stained with DAPI reagent. The slides were analyzed with a Leica TCS SP5 confocal microscope (Leica, Germany).

### Western blot

Cells were homogenized in radioimmunoprecipitation assay (RIPA) lysis buffer for the western blot analysis. Lysates (20 µg) were run on 10% SDS polyacrylamide gel at 100 V for 2 hours and transferred to a microporous polyvinylidene difluoride (PVDF) membrane at 100 mA for 2 hours. The membrane was blotted with goat polyclonal OGG-1 antibodies (1:1000)(CAT. # sc-12076), mouse polyclonal actin antibodies (1:1000)(CAT. # sc-8432), mouse polyclonal NFκB antibodies (1:1000) (CAT. # sc-8414), and phospho- NFκB rabbit polyclonal antibodies (Ser311 of p65, 1:1000) (CAT. # sc-166748) (Santa Cruz Biotechnology, Inc.) and processed via enhanced chemiluminescence (Pierce) [21].

### Statistical analysis

The data are expressed as the mean ± standard error. The statistical analysis was performed using ANOVA (Tukey's post hoc) or Student's *t*-test and the level of significance was defined as *P*<0.05 between any 2 groups. The data were analyzed using SPSS 13.0 software.

## Results

### Description of asthma with severe asthma

The clinical characteristics of asthma with severe asthma exacerbation are presented in Table 1. The mean age and gender of the patients with severe asthma exacerbation and V-D-deficiency (vitamin D3 <30 ng/ml) or V-D-sufficiency (vitamin D3>30 ng/ml) were not significantly different from the mean age and gender of the normal controls. Severe asthma exacerbation showed increased peripheral white blood cell counts and neutrophils compared to those in the normal controls (*p = 0.001*). Severe asthma exacerbation with V-D-deficiency or V-D-sufficiency showed higher PCO_2_ and lower PO_2_; however, there was no difference in the PCO_2_ and PO_2_ between severe asthma exacerbation with V-D-deficiency and V-D-sufficiency (Table 1, *p = 0.067*). Severe asthma exacerbation with V-D-deficiency did not show a difference in the endotoxin level compared to that in severe asthma exacerbation with V-D-sufficiency (Table 1, *p = 0.056*).

**Table 1:** Characteristics of severe asthma exacerbation.

	control	vitamin D>30ng/ml	vitamin D>30ng/ml
sample	16	16	16
Age(year)	46±4	45±15	50±10
Sexes	8F 8M	9F 7M	8F 8M
Leucocyte10*9	7.5±1.8	11.2±4	12.97±4.7
Neutrophils10*9	4.7±1.0	8. 9±4.5	10.6±4.4
Lymphocyte10*9	2.0±1.7	1.5±1.0	1.3±0.5
Eosinophils 10*9	0.25±0.04	0.20±0.09	0.19±0.13
PO2(mmHg)		57±10	53±22
PCO2(mmHg)		56±6	60±12
PH		7.4±0.03	7.32±0.06
Endotoxin(pg/ml)		19.15±4.95	21.91±7.06
FEV1%	92.56±9.14	42.5±3.98	32.25±4.02

*Statistically significant (P ≤ 0.05). F for female, and M for male.

### V-D-deficiency enhances oxidative stress and DNA damage in severe asthma exacerbation

**Figure 1:**
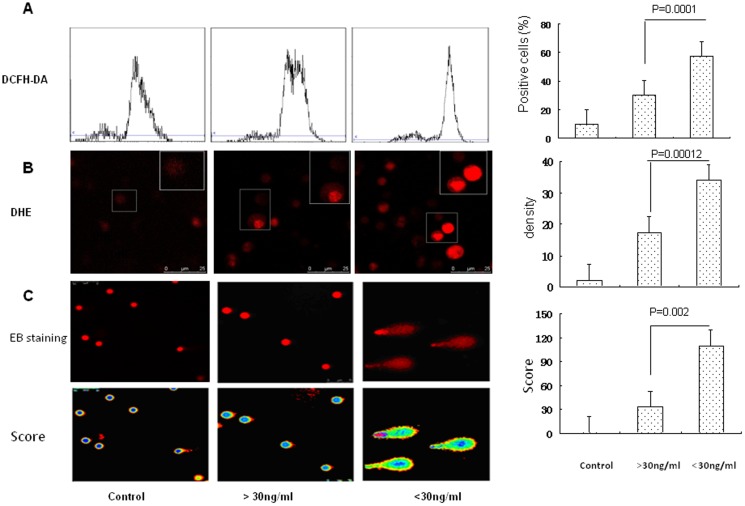
Oxidative stress and DNA damage in severe asthma exacerbation. A: The intracelluar ROS levels in PBMCs from the normal controls, severe asthma exacerbation and V-D-deficiency (vitamin D3 <30 ng/ml) or V-D-sufficiency (vitamin D3>30 ng/ml) were detected by flow cytometry using DCFH-DA, which is oxidized to DCF in the presence of ROS. The ROS level was represented by the percentage of ROS positive cells. B: The intracellular ROS levels in PBMCs from these asthmatic participants were detected by a confocal microscope using a DHE probe. The ROS level in PBMCs from these asthmatic participants was represented by the fluorescence intensity (X400). C: The DNA damage in PBMCs from these asthmatic participants was detected by comet assay using a confocal microscope(X400). The DNA damage score was analyzed with **Comet Assay IV** software.

The effects of oxidative stress on asthma remain unclear. ROS level and DNA damage in PBMCs from the normal controls, severe asthma exacerbation and V-D-deficiency or V-D-sufficiency were directly measured.Using an ROS probe, we found that severe asthma exacerbation exhibited increased ROS in PBMC compared to that in the normal controls. V-D-deficiency showed increased ROS-positive cells and ROS density in severe asthma exacerbation compared to that in V-D-sufficiency (vitamin D>30 ng/ml) (Figure 1A and B, *p = 0.0001 and 0.00012,n = 16*). Additionally, DNA damage in PBMC was detected by comet assay. DNA damage in PBMC exhibited differential increases in severe asthma exacerbation. A total damage score for each slide in V-D-deficiency was significantly increased compared to the scores in V-D-sufficiency (Figure 1C, *p = 0.002,n = 16*). These results indicate that V-D-deficiency shown increased oxidative stress and DNA damage in severe asthma exacerbation.

### V-D-deficiency showed lower FEV1%, SOD and increased TNF-α, NFκB in severe asthma exacerbation

**Figure 2:**
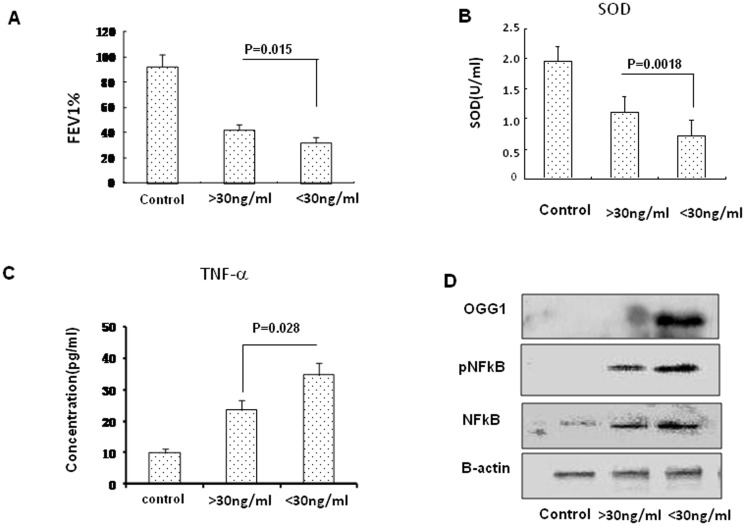
FEV1%, SOD activity, TNF-α, OGG1 and NFκB in severe asthma exacerbation. A: FEV1% in severe asthma exacerbation with V-D-deficiency and V-D-sufficiency. B: The serum SOD activity was measured using an SOD assay kit. C: Standard ELISA was performed to determine the levels of TNF-α in serum. D: OGG1 and NFκB in PBMC from severe asthma exacerbation with V-D-deficiency and V-D-sufficiency were analyzed by western blot. β-actin was used as the loading controls. The data are the means ± SEM.

To reflect the physiological relevance of Vitamin D deficiency, we evaluated FEV1%. We found that FEV1% was significantly decreased in severe asthma exacerbation compared to the normal controls (. V-D-deficiency patients (n = 16) showed lower FEV1% in severe asthma exacerbation compared to V-D-sufficiency patients (n = 16) (p = 0.015, Figure 2A). It was demonstrated that SOD is inactivated in bronchial brush material obtained from patients with mild asthma [22]. In this study, the serum SOD value in V-D-deficiency (n = 16) was significantly decreased compared to that in V-D-sufficiency (*p = 0.0018*, Figure 2B). The results indicate that V-D-deficiency aggravated the oxidative milieu changes in severe asthma exacerbation. TNF-α is a critical proinflammatory cytokine that might play an important role in severe refractory disease [23]. In our studies, serum TNF-α in V-D-deficiency in severe asthma exacerbation (n = 16) was significantly increased compared to that in V-D-sufficiency (n = 16) (*p = 0.028*, Figure 2C). Severe asthma exacerbation increased the expression and phosphorylation of NFκB in V-D-deficiency and V-D-sufficiency compared to that in the normal controls. V-D-deficiency increased the expression and phosphorylation of NFκB in severe asthma exacerbation compared to that in V-D-sufficiency (Figure 2D). Additionally, 8-Oxoguanine-DNA glycosylase (OGG-1) is a base excision DNA repair enzyme associated with oxidative stress damage [21]. In this study, V-D-deficiency increased the OGG1 expression in severe asthma exacerbation compared to that in V-D-sufficiency (Figure 2D). These results indicate that V-D-deficiency is associated with increased expression levels of TNF-α, OGG1 and NFκB as well as NFκB phosphorylation.

### V-D-deficiency decreased the corticosteroid response in severe asthma exacerbation

**Figure 3:**
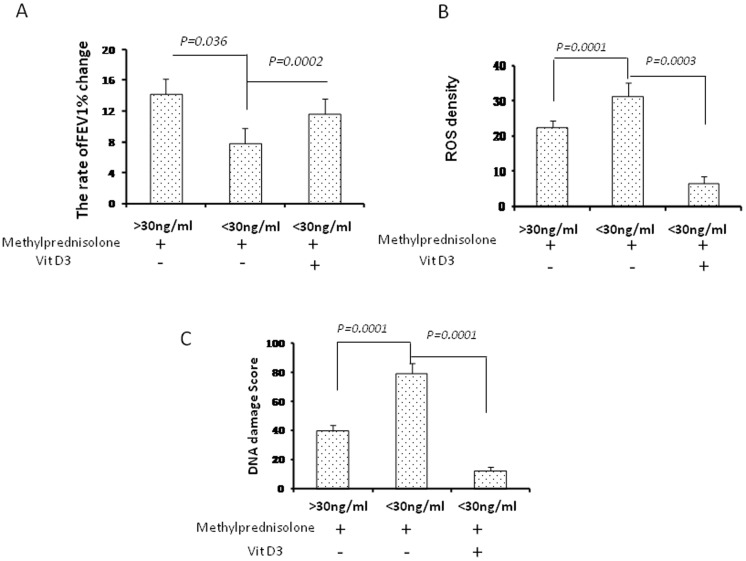
The change in FEV1%, ROS and DNA damage in severe asthma exacerbation treated with methylprednisolone and vitamin D3. A: The rates of the FEV1 change in severe asthma exacerbation treated with methylprednisolone and vitamin D3. B: The intracellular ROS in PBMCs from these asthmatic participants were detected by confocal microscopy using a DCFH-DA probe. The ROS levels were represented by the fluorescence intensity. The quantification of the ROS positive cell density in 10-random cell fields containing 500 cells(X400). C: The DNA damage in PBMCs from these asthmatic participants was detected by comet assay using confocal microscopy (X400). The DNA damage score was analyzed with **Comet Assay IV** software.

Oxidative stress contributes to the low response to glucocorticoids through the down-regulation of histone deacetylase (HDAC) activity [24]. In this study, the asthma patients with severe asthma exacerbation were treated with 80 mg/day of methylprednisolone for 7 days. The asthma patients with V-D-deficiency in V-D supplement (n = 8) were additionally injected with 7.5 mg vitamin of D3 at day 1 and 4. The FEV1%, ROS levels and DNA damage were detected after 7 days. The rates of FEV1 change in the V-D-deficiency patients treated with methylprednisolone (no V-D supplement,n = 8) were significantly lower than those in the V-D-sufficiency patients treated with methylprednisolone and vitamin D3 (n = 8) (p = 0.036, Figure 3A). These results demonstrated that V-D-deficiency decreased the corticosteroid response in severe asthma exacerbation. Vitamin D3 administration significantly increased the rates of FEV1 in the V-D-deficiency patients treated with methylprednisolone (V-D supplement, n = 8) (*p* = 0.0002, Figure 3A). These results indicate that vitamin D3 improved the response to methylprednisolone in severe asthma exacerbation with V-D-deficiency. The ROS levels in the V-D-deficiency patients treated with methylprednisolone were significantly higher than those in the V-D-sufficiency patients treated with methylprednisolone (Figure 3B, *p = 0.0001*). The ROS levels were significantly decreased in the V-D-deficiency patients treated with methylprednisolone and vitamin D3 (V-D supplement, n = 8) compared to the ROS levels in the V-D-sufficiency patients treated with methylprednisolone (no V-D supplement,n = 8) (Figure 3B, *P* *= 0.0003*). The DNA damage scores in the V-D-deficiency patients treated with methylprednisolone were significantly increased, compared to the DNA damage scores of the V-D-sufficiency patients treated with methylprednisolone (Figure 3C, *P* *= 0.0001*). In the V-D-deficiency asthma patients treated with methylprednisolone, the DNA damage scores in the patients treated with vitamin D3 (V-D supplement, n = 8) were significantly decreased compared to the patients treated without vitamin D3 (no V-D supplement, n = 8) (Figure 3C, *P* *= 0.0001*). These data suggest that supplemental vitamin D3 decreased oxidative stress in severe asthma exacerbation with V-D deficiency

### Vitamin D3 inhibited LPS-induced oxidative stress and TNF-α and NFκB in airway epithelial cells

**Figure 4:**
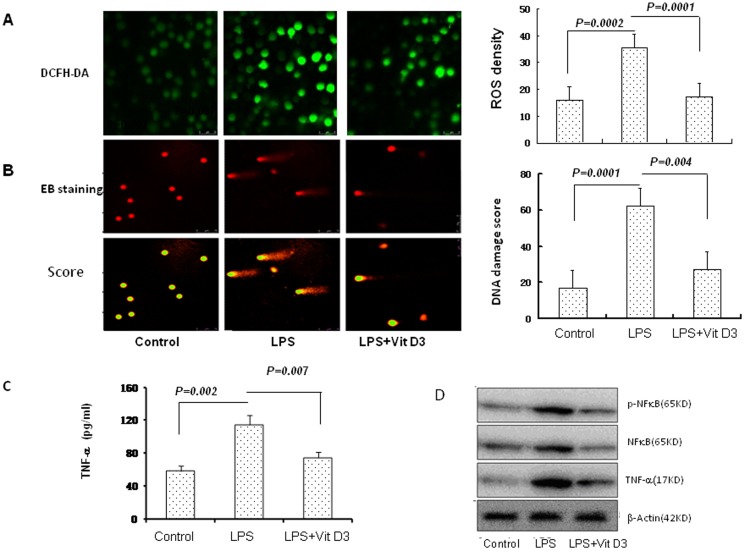
The effect of vitamin D3 on oxidative stress, DNA damage, TNF-α and NFκB in LPS-primed airway epithelial cells. The ROS and DNA damage in airway epithelial cells was measured in the presence or absence of vitamin D3 and treated with LPS for 24 hours. A: ROS was detected by confocal microscopy using a DCFH-DA probe. The ROS level was represented by fluorescence intensity (X400). B: The DNA damage was measured by comet assay. The DNA damage score was analyzed with **Comet Assay IV** software. **C**: Standard ELISA was performed to determine the levels of TNF-α. D: NFκB were analyzed by western blot. β-actin was used as the loading controls.

High endotoxin levels have been detected in bronchoalveolar lavage (BAL) fluid from subjects with corticosteroid-resistant (CR) asthma. LPS exposure contributes to CR asthma [8]. To assess the effect of vitamin D3 on LPS-induced oxidative stress and DNA damage, airway epithelial cells (16HBE) were stimulated with LPS for 24 hours, and vitamin D3 was added. Using a ROS probe, we found that LPS prime increased the ROS-positive cells compared to the control cells, and vitamin D3 decreased the ROS-positive cells, compared to the LPS-stimulated cells (Figure 4A). LPS caused oxidative stress. The DNA damage scores in the LPS-stimulated cells were significantly increased compared to that in the control cells. The vitamin D3 supply decreased the DNA damage scores compared to that in the LPS-stimulated cells (without vitamin D3) (Figure 4B). TNF-α was significantly increased in the LPS-stimulated cells compared to in the control cells. Vitamin D3 inhibited TNF-α in the LPS-stimulated cells (Figure 4C). The effect of vitamin D3 on LPS–induced NFκB remains unclear. Figure 4D shows that 10 µg/ml LPS significantly increased the expression and phosphorylation of NFκB, and vitamin D3 inhibited the LPS-induced expression and phosphorylation of NFκB (Figure 4D).

### Vitamin D3 inhibited H_2_O_2_-induced oxidative stress and increased the nuclear translocation of the glucocorticoid receptors

**Figure 5:**
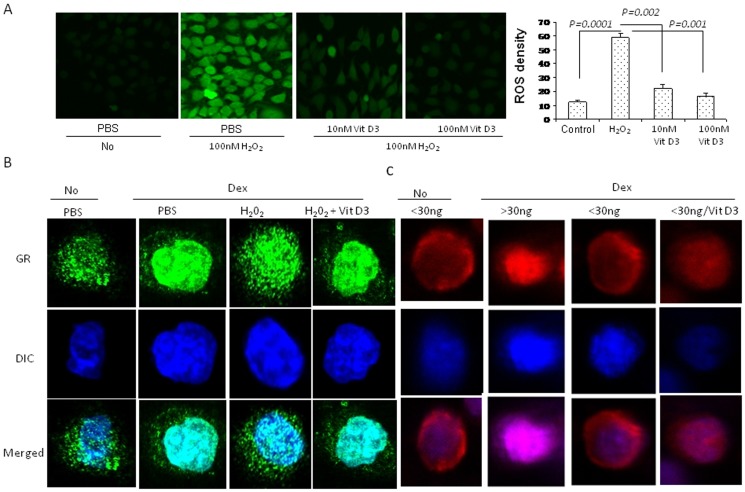
The effect of vitamin-D3 on oxidative stress and the nuclear translocation of the glucocorticoid receptor. A: The ROS in airway epithelial cells was detected by confocal microscopy using a DCFH-DA probe. The ROS level was represented by fluorescence intensity (X400).B: The nuclear translocation of GR was assessed by immunocytochemistry of the airway epithelial cells treated with Dex for 30 minutes. FITC- conjugated anti-rabbit secondary Abs (X400). C: The PBMC in severe asthma exacerbation with V-D-deficiency or V-D-sufficiency were incubated with or without vitamin D3 for 12 hours. The nuclear translocation of GR was assessed by immunocytochemistry of the PBMC treated with Dex for 30 minutes. TRITC–conjugated anti-rabbit secondary Abs (X400).

Glucocorticoid receptor (GR) nuclear translocation play a critical role in glucocorticoid therapy [25]. However, 1, 25(OH) 2 D3 impacts GR nuclear translocation in asthma remains unclear. To assess the effect of vitamin D3 on GR nuclear translocation in H_2_O_2_-induced oxidative stress, airway epithelial cells (16HBE) were stimulated with H_2_O_2_ for 1 hours in the presence or absence of 1, 25(OH) 2D3. Treatment of 16HBE cells with 100nM µg H_2_O_2_ induced ROS production as compared to untreated 16HBE cells with 1, 25(OH) 2 D3. Pretreatment of 16HBE cells with 10nM and 100nM 1, 25(OH) 2 D3 for 1 hours caused a significant decrease in ROS in H_2_O_2_-stimulated 16HBE cells as compared to untreated 16HBE cells with 1, 25(OH) 2 D3 (Figure 5A). It have been confirmed that H2O2 inhibited the ligand-stimulated nuclear translocation of glucocorticoid receptor [26]. The effect of 1, 25(OH) 2 D3 on GR nuclear translocation in H_2_O_2_-stimulated cells has not been demonstrated. In our studies, H_2_O_2_ decreased GR nuclear translocation in 16HBE cells following dexamethasone stimulation, which induced GR nuclear translocation. 1, 25-hydroxyvitamin D3 pretreatment enhanced the dexamethasone induced-GR nuclear translocation in H_2_O_2_-stimulated cells (Figure 5B). To evaluate the clinical relevance of cellular studies, we measured GR nuclear translocation in monocytes from V-D deficiency and V-D-sufficiency severe asthma patients. Our studies found that V-D-sufficiency asthma patients showed normal GR nuclear translocation in monocytes following dexamethasone stimulation. The dexamethasone induced-GR nuclear translocation in monocytes in the V-D deficiency severe asthma patients was significantly decreased compared to that in the V-D-sufficiency asthma patients (Figure 5C). In the monocytes from the V-D-deficiency asthma patients, the 1, 25-hydroxyvitamin D3 pretreatment enhanced the dexamethasone induced-GR nuclear translocation (Figure 5C).

## Discussion

Respiratory infections such as viruses, chlamydia and mycoplasma might cause wheezing and acute exacerbation of asthma; however, bacterial infection-induced acute exacerbation of asthma remains undefined [27], [28]. Bacterial colonization of the lower airways has been reported to be typical in patients with chronic severe asthma. One study reports that 52% of chronic severe asthma cases showed positive sputum cultures, predominantly with *H influenzae, P aeruginosa, S aureus* and *S pneumoniae strains* [29]. Vitamin D deficiency predisposes to viral respiratory tract infections. A lack of vitamin D suppresses adaptive immunity and increases the risk and severity of asthma [30]. In this study, acute exacerbations of asthma with a radiological diagnosis of pneumonia were excluded from the study. The patients with acute exacerbation of asthma exhibited more pronounced blood neutrophils. V-D-deficiency in acute exacerbation of asthma showed significantly increased blood neutrophils. The results suggested that V-D-deficiency may be associated with the phenotype of neutrophilic inflammation in asthma exacerbation.

Oxidative stress has been recognized as contributing significantly to the inflammatory pathology of bronchial asthma. Dunstan et al., found that higher antioxidant levels were not associated with reduced allergen responsiveness in allergic adults [31]. The serum total antioxidant status was correlated with the severity criteria, and measurement of the total antioxidant status could be useful to evaluate asthma attacks [32]. Insufficient 25(OH) D levels increased oxidative/nitrosative stress, inflammation, and endothelial activation in severely obese children. Supplemental vitamin D3 decreased oxidative DNA damage in normal human colorectal mucosa [33], [34]. The activity of SOD is as an intracellular antioxidant enzyme. SOD activity in the asthmatic patients was lower than that in the healthy controls [14]. The mechanism of oxidative stress in asthma remains unclear. In this study, the patients with severe asthma exacerbation showed increased ROS, DNA damage and OGG1 in the peripheral blood mononuclear cells. SOD in V-D-deficiency was significantly decreased compared to SOD in V-D-sufficiency in severe asthma exacerbation. The results indicated that V-D-deficiency aggravated the oxidative milieu changes in severe asthma exacerbation. V-D-deficiency increased oxidative stress and DNA damage in severe asthma exacerbation, and oxidative stress plays a key role in the pathophysiology of severe asthma exacerbation.

Oxidative stress is an important factor in corticosteroid insensitivity by the inhibition of HDAC-2 activity and expression. Antioxidants might be shown to be beneficial in restoring corticosteroid function [35]. The proposed extrarenal effects of 25-hydroxyvitamin D3 include increased antimicrobial peptide production, regulation of the inflammatory response, and airway remodeling [36]. Additionally, vitamin D deficiency is associated with poorer lung function. Low vitamin D levels are associated with severe asthma exacerbation and decreased airway responsiveness in asthma [6], [37]. In this study, a significant negative association was present between V-D-deficiency and corticosteroid response in severe asthma exacerbation, with severe asthma exacerbation with V-D-deficiency showing a decreased corticosteroid response. V-D-deficiency in acute exacerbation of asthma showed significantly a decreased FEV1%. FEV1% was significantly decreased in severe asthma exacerbation with V-D-deficiency compared to that in V-D-sufficiency. These observations indicated that the vitamin D status is related to a decreased FEV1% in severe asthma exacerbation. The FEV1 changes in severe asthma exacerbation with V-D-deficiency were significantly lower than those with V-D-sufficiency during methylprednisolone therapy. Supplemental vitamin D was beneficial for increased corticosteroid response and decreased oxidative stress. This study demonstrated an association between V-D-deficiency and corticosteroid response. The association between vitamin D and oxidative stress is supported by *in vivo* studies. Supplemental vitamin D significantly decreased oxidative stress and DNA damage in severe asthma exacerbation with V-D-deficiency.

TNF-α plays an important role in severe refractory asthma [23]. Kox et al., reported that the plasma levels of vitamin D are not correlated with the LPS-induced TNF-α, IL-6 and IL-10 cytokine response in humans during experimental human endotoxemia [38]. However, Di Rosa et al., found that vitamin D facilitated the expression of inflammatory mediators such as IL-1β, IL-6 and TNF-α in human monocytes/macrophages [39]. Additionally, TNF-α mediated the vitamin D receptors. TNF-α-stimulation significantly decreased the expression and activity of vitamin D receptors in porcine coronary artery smooth muscle cells [40]. We found that severe asthma exacerbation with V-D-deficiency showed increased TNF-α, NFκB expression and NFκB phosphorylation compared to that with 25-hydroxyvitamin D-sufficiency.

A previous study confirmed that LPS-treated animals developed oxidative stress, and LPS-induced inflammatory responses were associated with oxidative stress. Some studies demonstrate that LPS-stimulated macrophages increased the production of TNF-α. The LPS-induced overexpression of inflammatory mediators in vascular smooth muscle cells was via the TLR4/MAPK/NFκB pathways [41], [42]. A central event in the inflammatory response to LPS is the activation of transcription factor NFκB [43], [44]. In our present study, TNF-α was significantly increased in the LPS-stimulated cells. Additionally, LPS induced NFκB expression and phosphorylation. In our studies, our results show that LPS-stimulated airway epithelial cells caused increased ROS release and DNA damage. Supplemental vitamin D decreased the ROS release and DNA damage in LPS-stimulated airway epithelial cells. Vitamin D3 inhibited the LPS-induced expression of TNF-α, NFκB expression and NFκB phosphorylation. These results suggest that Vitamin D3 is a potential anti-inflammatory agent by attenuating the generation of TNF-α and blocking ROS generation and NFκB activation pathways in LPS-stimulated phagocytes.

Steroids could not contain the increased levels of T_H_17 cytokines in steroid-resistant asthma; however, 1, 25(OH) 2D3 inhibited T_H_17 cytokine production in all the patients [45] and demonstrated anti-inflammatory and corticosteroid-enhancing effects in the monocytes of asthma patients. The vitamin D3 pretreatment enhanced dexamethasone-induced GR binding and histone acetylation in monocytes from asthma [46]. Oxidative stress inhibited HDAC2 activity and expression through activation of phosphoinositide 3-kinase δ, which is a molecular mechanism of the steroid resistance in asthmatic patients [47]. In our present studies, H_2_O_2_ decreased GR nuclear translocation in 16HBE cells following dexamethasone stimulation. 1, 25-hydroxyvitamin D3 pretreatment enhanced the dexamethasone induced-GR nuclear translocation in H_2_O_2_-stimulated cells. V-D-deficiency asthma patients showed decreased nuclear translocation of glucocorticoid receptors in monocytes following dexamethasone stimulation. We used 1, 25-hydroxyvitamin D3 to pretreat monocytes from V-D-deficiency asthma patients and found that 1, 25-hydroxyvitamin D3 increased nuclear translocation of glucocorticoid receptors in monocytes following dexamethasone stimulation. Our studies represent a novel vitamin D mechanism in severe asthma exacerbation with V-D-deficiency.

This study of severe asthma exacerbation with V-D-deficiency showed oxidative stress and DNA damage in peripheral blood monocytes. V-D-deficiency increased the oxidative stress and DNA damage in exacerbation of severe asthma. Severe asthma exacerbation with V-D-deficiency increased TNF-α, NFκB expression and NFκB phosphorylation. Supplemental vitamin D increased the corticosteroid response and decreased oxidative stress. Supplemental vitamin D decreased ROS release and DNA damage in LPS-stimulated airway epithelial cells. Vitamin D3 down-regulates the expression of TNF-αand NFκB as well as NFκB phosphorylation in LPS-stimulated airway epithelial cells, suggesting a possible mechanism for vitamin D3 therapy in severe asthma exacerbation. Our studies suggest that V-D-deficiency intensifies oxidative stress and DNA damage, suggesting a possible mechanism for corticosteroid resistance in severe asthma exacerbation.
